# The Internet‐Based Conversational Engagement Trial (I‐CONECT) for Socially Isolated Older Adults: Conceptual Framework and Latest Findings

**DOI:** 10.1002/alz70860_105335

**Published:** 2025-12-23

**Authors:** Hiroko H Dodge, Kexin Yu, Liu Chen, Chao‐Yi Wu

**Affiliations:** ^1^ Massachusetts General Hospital, Harvard Medical School, Boston, MA, USA; ^2^ University of Texas Southwestern Medical Center, Dallas, TX, USA

## Abstract

**Background:**

Social isolation is a known risk factor for dementia, and addressing it may help delay cognitive decline. The Internet‐based Conversational Engagement Clinical Trial (I‐CONECT; www.i‐conect.org; ClinicalTrials.gov: NCT02871921) is the latest in a series of NIH‐funded studies over the past 15 years, focusing on conversational interactions as a cognitive stimulator. Unlike prior studies, I‐CONECT specifically targeted socially isolated older adults with limited opportunities for conversation, expecting the greatest impact in this population. This presentation will overview the study, its conceptual framework, and recent findings following the publication of topline results in 2023. New findings highlight the intervention's clinical relevance, including time‐saved analysis and its impact on social behavior and speech characteristics.

**Methods:**

186 socially isolated participants aged 75+ (MCI: *N* = 100, Normal Cognition: *N* = 86) were recruited from the community and randomized in I‐CONECT. The experimental group engaged in 30‐minute semi‐structured video chats with trained staff 4 times per week for 6 months (induction), followed by twice per week for an additional 6 months (maintenance). Both groups received a weekly 10‐minute check‐in phone call to monitor changes in health and social activities. Unique features of the trial included standardized yet natural conversations using stimulating picture prompts, staff training and user‐friendly devices.

**Results:**

New findings since the 2022 AAIC presentation include: (1) The top 30% of responders delayed cognitive decline by 6 months or more in global cognitive function based on individual treatment response (ITR) analyses, (2) Participants in the MCI experimental group were more likely to contact friends over time compared to the control group, suggesting that the I‐CONECT intervention could stimulate weekly social interactions among socially isolated older adults, potentially providing additional cognitive stimulation, (3) Digital twin modeling approaches confirmed the robustness of our efficacy using an alternative control group derived from an observational study, and (4) Changes in speech characteristics were observed in the experimental group.

**Conclusions:**

These recent results provide further evidence of the positive impact of social interactions on cognition and daily functioning in socially isolated older adults. They reinforce previous findings and highlight the potential of internet‐based, conversation‐focused interventions to support cognitive health and improve quality of life.

